# Successful Diagnoses and Remarkable Metabolic Disorders in Patients With Solitary Hypothalamic Mass: A Case Series Report

**DOI:** 10.3389/fendo.2021.693669

**Published:** 2021-09-16

**Authors:** Boni Xiang, Quanya Sun, Min He, Wei Wu, Bin Lu, Shuo Zhang, Zhaoyun Zhang, Yehong Yang, Yiming Li, Yue Wu, Zhenwei Yao, Haixia Cheng, Li Pan, Qing Miao, Yongfei Wang, Hongying Ye

**Affiliations:** ^1^Department of Endocrinology and Metabolism, Huashan Hospital, Fudan University, Shanghai, China; ^2^Department of Radiology, Huashan Hospital, Fudan University, Shanghai, China; ^3^Department of Pathology, Huashan Hospital, Fudan University, Shanghai, China; ^4^Department of Neurosurgery, Huashan Hospital, Fudan University, Shanghai, China; ^5^Shanghai Gamma Hospital, Fudan University, Shanghai, China

**Keywords:** hypothalamus, hypopituitarism, metabolic disorders, hypothalamic obesity, stereotactic biopsy

## Abstract

**Background:**

Solitary intracranial hypothalamic mass occurs rarely. The etiological diagnosis of solitary hypothalamus lesion is challenging and often unachievable. Although previous studies indicated that lesions affecting the hypothalamus often cause significant metabolic disorders, few reports about the metabolic disturbances of patients with solitary hypothalamic mass have been reported.

**Method:**

Twenty-five patients with solitary hypothalamus lesions who had been evaluated and treated in Huashan Hospital from January 2010 to December 2020 were retrospectively enrolled. The clinical manifestations, radiological features, endocrine and metabolic disorders, and pathology were analyzed.

**Results:**

The male to female ratio was 5/20. The median age of onset was 22 (19, 35) years old. The most common initial symptom was polydipsia/polyuria (19/25, 76.0%) and amenorrhea (9/20, 45.0%). A high prevalence of hypopituitarism of different axes was found, with almost all no less than 80%. Central hypogonadism (21/22, 95.5%) and central diabetes insipidus (19/21, 90.5%) were the top two pituitary dysfunctions. Conclusive diagnoses were achieved by intracranial surgical biopsy/resection or stereotactic biopsy in 16 cases and by examining extracranial lesions in 3 cases. The pathological results were various, and the most common diagnoses were Langerhans cell histiocytosis (7/19) and hypothalamitis (5/19). The mean timespan from onset to diagnosis in the 19 cases was 34 ± 26 months. Metabolic evaluations revealed remarkable metabolic disorders, including hyperlipidemia (13/16, 81.3%), hyperglycemia (10/16, 62.5%), hyperuricemia (12/20, 60%), overweight/obesity (13/20, 65.0%), and hepatic adipose infiltration (10/13, 76.6%).

**Conclusion:**

Either surgical or stereotactic biopsy will be a reliable and relatively safe procedure to help to confirm the pathological diagnosis of solitary hypothalamic mass. Metabolic disorders were severe in patients with solitary hypothalamic mass. The management of such cases should cover both the treatment of the primary disease, as well as the endocrine and metabolic disorders

## Introduction

Hypothalamus is one of the essential regions of the brain and acts as the regulatory center of the endocrine system. It plays vital roles in regulating endocrine functions, water metabolism, food intake, body weight, body temperature, sleep/wake cycle, and emotions (1). Patients with hypothalamic or peri-hypothalamic lesions may present with similar hormonal or neurologic disorders due to mass effect upon the hypothalamus or direct invasion of this diencephalic region. The etiology of hypothalamic lesions is various, includes congenital developmental malformations, primary tumors, metastatic tumors, hemangioma, inflammatory and granulomatous diseases, trauma, infections, etc. (1). Cases of intracranial solitary hypothalamic mass are rarely reported. Such patients, despite different etiologies, can have similar clinical manifestations and radiological features. The etiological diagnosis of solitary hypothalamus lesion is crucial to determine the treatment. But it is very challenging in practice and often unachievable. Few cases about intracranial solitary hypothalamus mass with a confirmed pathological diagnosis were reported (2, 3). Considering the vital role in regulating metabolism and energy balance, patients with hypothalamic lesions tend to have metabolic disorders. However, few reports have addressed the subject comprehensively. To explore the diagnosis procedures and to reveal the endocrine and metabolic consequences of solitary hypothalamus lesions, we present a case series report of 25 patients with intracranial solitary hypothalamic mass.

## Method

This is a retrospective study including 25 patients with intracranial solitary hypothalamic lesions admitted to Huashan Hospital from January 2010 to December 2020. The clinical manifestations, endocrine and metabolic disorders, and pathology were reviewed.

### Radiological Examinations

Magnetic resonance imaging (MRI) was performed in all patients, using a 3-T scanner (Signa; GE Medical Systems, Shanghai, China). Precontrast T1-weighted spin-echo images and T2-weighted fast spin-echo images were recorded, followed by contrast-enhanced T1-weighted imaging (T1WI). An intracranial solitary hypothalamic lesion is defined as the radiologically proved lesion within the anatomical area of the hypothalamus in MRI. The pre-operation/biopsy/treatment MRI images were reviewed by experienced radiologists, particularly focusing on the neuroradiological variables including size, shape, and consistency of the lesion, enhancement pattern, third ventricle floor status, degree of third ventricle expansion, displacement/atrophy of mammillary bodies, degree of pituitary stalk infiltration, presence of hydrocephalus, and optic chiasm invasion. The size of each lesion was measured at the greatest diameter in each of the three planes. Total-body 18-fluorodeoxyglucose positron emission tomography-CT (18-FDG-PET-CT) was performed in 9 cases, using a combined PET/CT scanner (Siemens Biograph Sensation 16; Siemens, Berlin, Germany).

### Surgery and Biopsy

Stereotactic hypothalamic biopsy in 6 patients, surgical biopsy through transcranial approaches or endoscopic endonasal approach in 10 patients, and surgical resection of the hypothalamus lesions in 4 patients were performed. The detailed procedure of stereotactic biopsy can be referred to in our published paper (4).

### Endocrine Evaluation

Endocrine functions were routinely evaluated in most patients. Blood and urine samples were obtained at 8:00 AM before any medication. Central adrenal insufficiency (CAI) was defined as a serum cortisol level <3 μg/dL or a peak serum cortisol level <18.1 μg/dL at 30 or 60 minutes in a corticotropin stimulation test (5). Central hypothyroidism (CHT) was defined as a free T4 level below the laboratory reference range in conjunction with a low, normal, or mildly elevated thyroid-stimulating hormone (5, 6). The diagnosis of central hypogonadism (CHG) was made based on low morning serum total testosterone levels with non-raised gonadotropin levels in men or a low serum estradiol level simultaneously low luteinizing hormone and follicle-stimulating hormone in women presenting oligomenorrhea or amenorrhea (7). Hyperprolactinemia (HPL) was defined as a serum PRL level above the laboratory reference range. Central diabetes insipidus (CDI) was confirmed by a water deprivation test followed by a desmopressin test. A urine osmolality of less than 300 mOsm/kg in the presence of fluid deprivation and subsequent rise of urine osmolality by no less than 50% after arginine vasopressin stimulation supported the diagnosis of CDI (7). Besides, for patients with significant polyuria (more than 50 mL/kg of body weight/24 hours), CDI could also be diagnosed by the presence of low urine specific gravity and a good response to a therapeutic trial of desmopressin (5, 7). Evaluation of the growth hormone (GH) axis only included the assessment of IGF-1 levels.

### Metabolic Evaluation

Metabolic evaluations, including body mass index (BMI), liver ultrasonography, fasting/postprandial blood glucose (FBG/PBG), HbA1c, lipid profile, and serum uric acid (SUA), were performed in some cases.

### Statistical Analyses

Normal distributed continuous variables were expressed as mean values ± standard deviation (SD). Differences between groups were estimated using the Fisher’s exact test or the Mann-Whitney test. Simple linear regression coefficients and multiple regression analysis were used to examine the correlation among parameters. SPSS software (version 21.0, SPSS Inc) was used to perform statistical analyses. A two-tailed P value <0.05 was considered statistically significant.

### Ethical Approval

The study was approved by the ethics committee of Huashan Hospital affiliated to Fudan University. Consent was obtained after a full explanation of the study purpose and procedures.

## Results

### Symptoms

**Figure 1:**
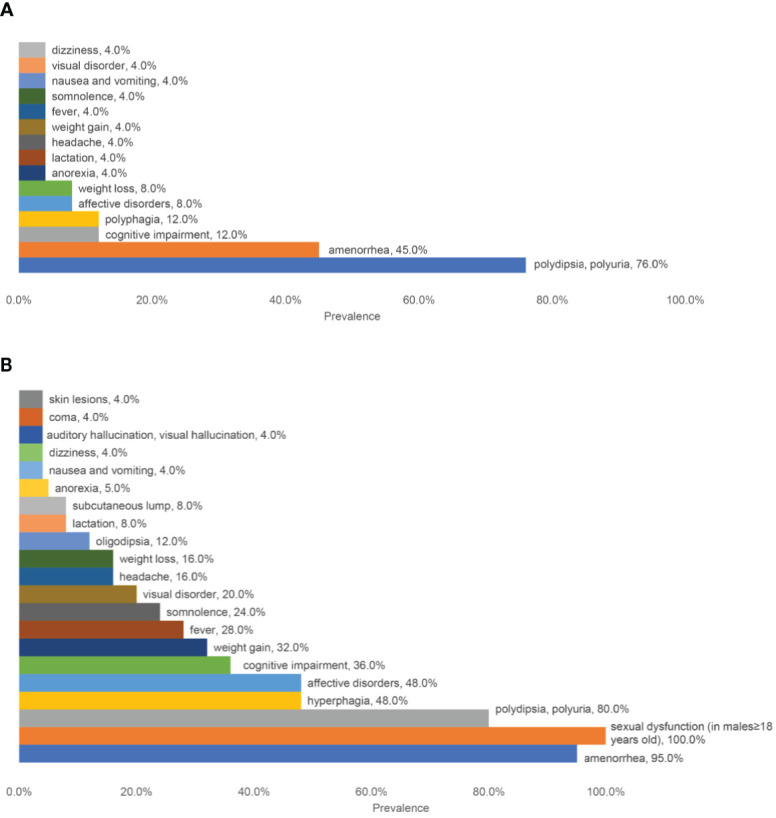
A summary of the prevalence of different symptoms. **(A)** The prevalence of initial symptoms; **(B)** The prevalence of all the symptoms.

A short description of patients was shown in **Table 1**. The male to female ratio was 5/20. The median age of onset was 22 (ranging from 10 to 70) years old. The most common initial symptoms (**Figure 1A**) were polydipsia and polyuria (19/25, 76.0%) and amenorrhea (9/20, 45%, Patient No.5 had had menopause before onset). Other symptoms at onset included hyperphagia (3/25), cognitive impairment (3/25, mainly memory deterioration together with reduced ability to solve problems), affective disorders (2/25, manifesting as irritability), weight loss (2/25), weight gain (1/25), anorexia (1/25), lactation (1/25), headache (1/25), fever (1/25), dizziness (1/25), visual disorder (1/25), somnolence (1/25), nausea and vomiting (1/25). With the progression of the disease, new manifestations showed up subsequently (**Figure 1B**). Altogether, amenorrhea in females (19/20, 95%) and sexual dysfunction in males (4/4, 100%) were the top two symptoms, followed by polydipsia and polyuria (20/25, 80.0%). Oligodipsia occurred in 3 cases after the onset of diabetes insipidus. Hyperphagia was seen in 12/25 cases, and weight gain in 8/25. Specially, one patient (No.4) had anorexia and weight loss in the beginning but had hyperphagia and weight gain afterward. Besides, affective disorders like irritability or mood swings and cognitive impairment were common, which were seen in 12/25 and 9/25 separately. Fever with no evidence of infection occurred in 7 cases. Some patients showed symptoms related to mass effects, such as headache (4/25), visual disorders (7/25), nausea, and vomiting (1/25). Disturbance of consciousness developed, such as somnolence (6/25) and even coma (1/25, No.23), which may be related to severe hypernatremia (over 160mmol/L). This patient complained of symptoms of auditory and visual hallucination, too. Subcutaneous lumps were seen in two cases (No. 20&21), who were diagnosed with Langerhans cell histiocytosis (LCH). Patient No. 21 had systemic skin lesions manifesting as itches, ulceration, and exudation.

**Table 1:** Short description of patients.

No	Gender	Age of Onset(y/o)	Initial symptoms	All symptoms	AI	CHT	CHG	HPL	Low IGF-1	CDI	Diagnosis	DfOtD (months)	Treatment	Follow-up period (months)	Outcome
1	F	38	polydipsia, polyuria	polydipsia, polyuria, amenorrhea	N^*^	N	Y^†^	Y	Y	Y	Unknown	/	Radiotherapy	4	Stable lesion
2	F	26	amenorrhea	amenorrhea, polyphagia, irritability, memory deterioration	N/A^‡^	Y	Y	Y	N/A	Y	Unknown	/	/	16	Stable lesion
3	F	25	polydipsia, polyuria, anorexia, weight loss	polydipsia, polyuria, anorexia, weight loss, depressed mood, memory deterioration, polyphagia, weight gain	N/A	N/A	N/A	N/A	N/A	N/A	LCH	66	Surgical resection	44	Stable lesion
4	F	17	irritability, memory deterioration, difficulty with problem-solving, polyphagia, weight gain	irritability, memory deterioration, difficulty with problem-solving, polyphagia, weight gain, amenorrhea	N/A	N/A	Y	N/A	N/A	N/A	Germinoma	15	Radiotherapy	39	Lesion shrinkage after radiotherapy and stable afterwards
5	F	58	polydipsia, polyuria	polydipsia, polyuria, fever	Y	Y	Y	Y	N	Y	LCH	48	Radiotherapy	25	Lesion shrinkage after treatment and stable afterwards
6	F	21	polydipsia, polyuria, somnolence	polydipsia, polyuria, somnolence, amenorrhea, polyphagia, weight gain	Y	Y	Y	Y	Y	Y	Unknown	/	Gamma knife radiosurgery	9	Lesion shrinkage after treatment and stable afterwards
7	M	34	polydipsia, polyuria	polydipsia, polyuria, sexual dysfunction	Y	Y	Y	Y	N	Y	Hypothalamitis	33	Methylprednisolone and azathioprine	47	Lesion shrinkage after treatment and stable afterwards
8	M	22	nausea, vomiting	nausea, vomiting, headache, sexual dysfunction	Y	Y	Y	Y	Y	Y	Germinoma	26	Radiotherapy and chemotherapy	25	Lesion shrinkage after treatment and stable afterwards
9	F	21	polydipsia, polyuria	polydipsia, polyuria, amenorrhea, visual disorder, irritability, memory deterioration, polyphagia, weight gain, fever, somnolence	Y	Y	Y	N/A	N/A	Y	Embryonic germ cell tumors	3	Radiotherapy and chemotherapy	110	Lesion shrinkage after treatment and stable afterwards
10	F	37	polydipsia, polyuria, amenorrhea, weight loss	polydipsia, polyuria, amenorrhea, weight loss, apathy	Y	Y	Y	N/A	Y	Y	LCH	15	Radiotherapy and chemotherapy	40	Lesion shrinkage after treatment, death of systemic disease spread afterwards
11	F	32	polydipsia, polyuria	polydipsia, polyuria, polyphagia, weight gain, fever, somnolence, oligodipsia, disorientation, loss of memory	Y	Y	Y	Y	N	Y	Hypothalamitis	29	Methylprednisolone	/	Lesion shrinkage, lost to follow-up afterwards
12	F	17	polydipsia, polyuria, amenorrhea, fever	polydipsia, polyuria, amenorrhea, fever, irritability, polyphagia, weight gain, somnolence, oligodipsia	Y	Y	Y	Y	Y	Y	Hypothalamitis	30	Methylprednisolone and azathioprine	/	Lost to follow-up
13	F	19	polydipsia, polyuria, amenorrhea	polydipsia, polyuria, amenorrhea	Y	Y	Y	Y	N	Y	Hypothalamitis	24	Diagnostic radiotherapy; Methylprednisolone and azathioprine after biopsy	12	Lesion shrinkage; sudden death of unknown reason afterwards
14	F	35	polydipsia, polyuria	polydipsia, polyuria, amenorrhea, irritability, polyphagia, weight loss	Y	Y	Y	Y	Y	Y	Granulosa cell tumor	77	/	/	Lost to follow-up
15	F	35	amenorrhea	polydipsia, polyuria, amenorrhea, lactation, fever, somnolence, apathy	N/A	Y	Y	Y	Y	Y	LCH	35	Gamma knife radiosurgery	25	Death
16	F	22	polydipsia, polyuria, amenorrhea, polyphagia	polydipsia, polyuria, amenorrhea, polyphagia	N/A	N	Y	N	N/A	Y	Rosai-Dorfman disease	53	Gamma knife radiosurgery	8	Lesion shrinkage; death of acute pancreatitis afterwards
17	M	20	polydipsia, polyuria, irritability, memory deterioration, polyphagia	polydipsia, polyuria, irritability, memory deterioration, polyphagia, sexual dysfunction	Y	Y	Y	Y	N/A	Y	Hypothalamitis	3	Dexamethasone, methylprednisolone and azathioprine	132	Lesion shrinkage after treatment and stable afterwards
18	M	70	polydipsia, polyuria	polydipsia, polyuria, sexual dysfunction	Y	Y	Y	Y	N	Y	Metastatic carcinoma from lung	4	/	/	Lost to follow-up
19	F	19	amenorrhea	polydipsia, polyuria, amenorrhea, irritability, memory deterioration, difficulty with problem-solving, polyphagia, weight gain, somnolence, oligodipsia	N/A	Y	Y	Y	N/A	N/A	Unknown	/	Radiotherapy, methylprednisolone and azathioprine	78	Lesion shrinkage in the beginning; death afterwards
20	F	10	polydipsia, polyuria	polydipsia, polyuria, amenorrhea, headache, weight gain, subcutaneous lump on the forehead	N	Y	Y	N	Y	N	LCH	77	Chemotherapy	56	Lesion shrinkage after treatment and stable afterwards
21	F	15	polydipsia, polyuria, amenorrhea	polydipsia, polyuria, amenorrhea, subcutaneous lump on the occiput, skin itches/ulceration/exudation	Y	Y	Y	Y	Y	Y	LCH	17	Chemotherapy	/	Lost to follow-up
22	F	22	polydipsia, polyuria, headache, loss of memory	polydipsia, polyuria, headache, loss of memory, amenorrhea, lactation	N/A	N/A	N/A	N/A	N/A	N/A	LCH	81	Chemotherapy	/	Lesion shrinkage after treatment and stable afterwards
23	F	34	polydipsia, polyuria	polydipsia, polyuria, amenorrhea, mood swings, polyphagia, fever, auditory hallucination, visual hallucination, coma	N/A	Y	N/A	Y	N/A	Y	Unknown	/	Gamma knife radiosurgery	14	No change after treatment
24	F	40	polydipsia, polyuria	polydipsia, polyuria, amenorrhea, mood swings, visual disorder, loss of memory, fever	Y	Y	Y	Y	Y	Y	Unknown	/	/	11	Stable lesion
25	M	14	visual disorder, dizziness	visual disorder, dizziness	N	N	N	Y	N	N	Pilocytic astrocytoma	3	Surgical resection	/	Lost to follow-up

AI, adrenal insufficiency; CHT, central hypothyroidism; CHG, central hypogonadism; HPL, hyperprolactinemia; CDI, central diabetes insipidus; DfOtD, duration from onset to diagnosis; LCH, Langerhans cell histiocytosis. N^*^: no. Y^†^: yes. N/A^‡^: not available.

The follow-up period refers to the time span from the treatment to the timepoint of the last follow-up/death. For those no treatment was given, the follow-up period refers to the time span from the first admission to the timepoint of last follow-up/death.

### Radiological Features

**Figure 2:**
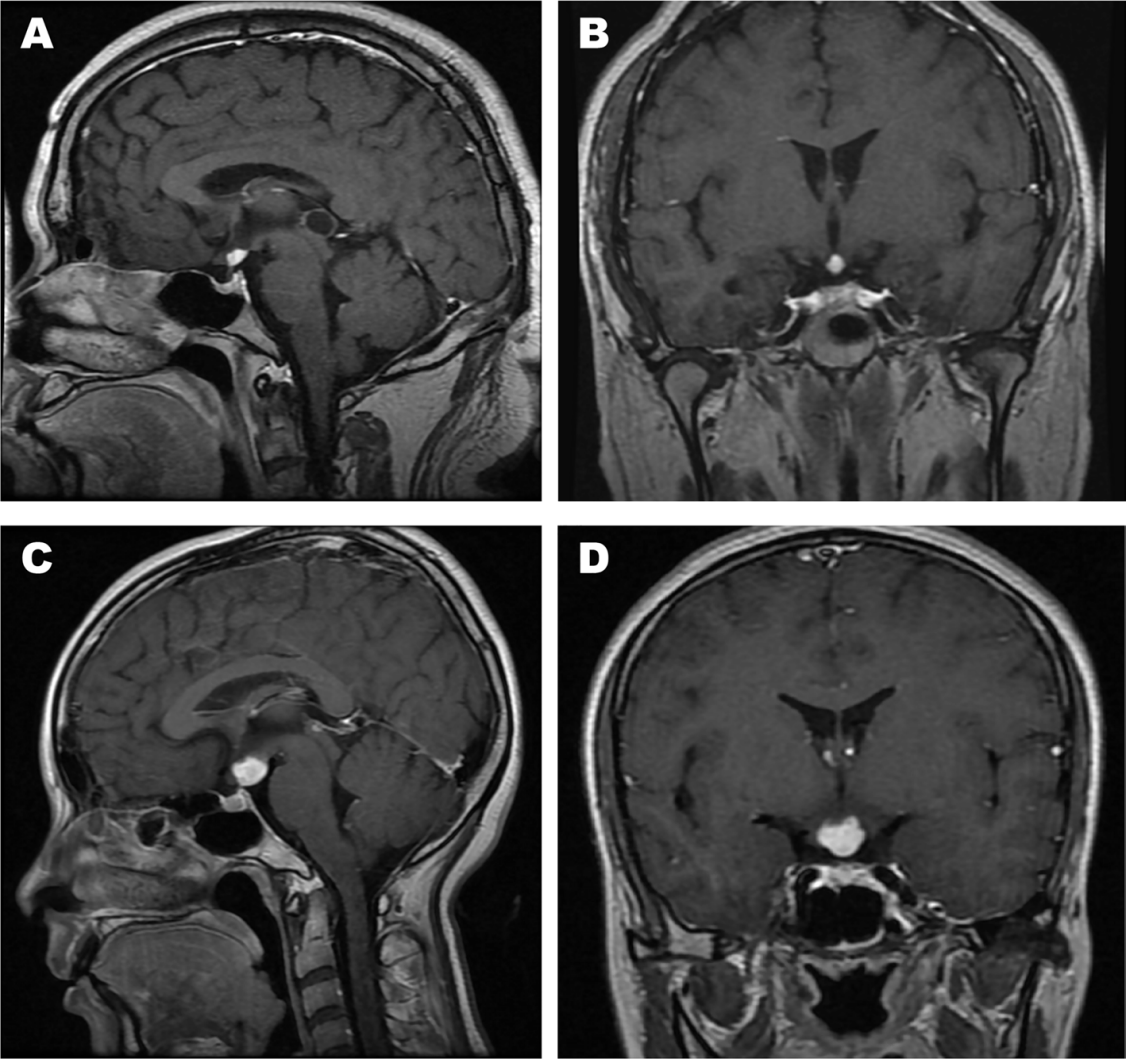
The T1-weighted postcontrast MR images of two typical cases. **(A)** Sagittal view of patient No. 7; **(B)** Coronal view of patient No. 7; **(C)** Sagittal view of patient No. 10; **(D)** Coronal view of patient No. 10.

**Table 2** shows a summary of the neuroradiological variables. The pre-operation/biopsy/treatment MR images of patients No. 2, 3, 18, 22, 25 were missing. The T1-weighted postcontrast coronal and sagittal views of MR images of two typical cases (patients No. 7 and 10) were shown in **Figure 2**. The MRI features mainly demonstrated hypointense or isointense T1WI and hyperintense T2WI for the hypothalamic lesions. In contrast-enhanced T1WI, all lesions showed enhancement patterns, either heterogeneous or homogeneous. Hydrocephalus was seen in 7 patients. In 5 (71.4%) of them, the lesions` maximum diameter in any of the three planes was no less than 2cm. The lesions took on an elliptical (6/20), round (7/20), polygonal (4/20), or lobulated (3/20) shape. A consistency of pure solid was seen in most cases (17/20), while mixed solid-cystic lesion was found in cases No. 4 and 9, and solid with small cystic change lesion in patient No. 11. Atrophy of mammillary bodies wasn`t found, but their displacement was observed in 8 cases, either downward (6/20) or upward (2/20). In 9/20 cases, the pituitary stalk was partially infiltrated, and in 3/20 wholly infiltrated. The bright signals in the posterior lobe of the pituitary were absent for all cases. Optic chiasm was partially infiltrated in 15/20 cases and wholly infiltrated in 3/20. Among the 18 patients, 6 had visual disorders. The third ventricle floor (TVF) identification was examined. The TVFs were completely identifiable in 3/20 cases, not visible in 10/20 patients, and in 7/20 cases, only mammillary bodies were visible. Third ventricle involvement was seen in 15/20 cases, with 1 wholly invaded and 14 partially invaded.

**Table 2:** Summary of neuroradiological variables.

No	Hydrocephalus	T1WI	T2WI	Size (cm)	Shape	Lesion consistency	Enhancement pattern	Displacement of MBs	Atrophy of MBs	Pituitary stalk	Optic chiasm invasion	Third ventricle involvement	TVF identification
1	N^*^	hypointensity	hyperintensity	1.2×1.3×0.9	elliptical	pure solid	marked homogeneous enhancement	Y^†^(downward)	N	partially infiltrated	partially infiltrated	partial invasion	only MBs visible
2	Images N/A^‡^												
3	Images N/A												
4	N	mild hypointensity	hyperintensity	2.1×2.1×2.5	lobulated	mixed solid-cystic	marked homogeneous enhancement	N	N	not affected	partially infiltrated	partial invasion	not visible
5	N	mild hypointensity	mild hyperintensity	0.8×1.3×1.2	elliptical	pure solid	marked homogeneous enhancement	Y(downward)	N	partially infiltrated	partially infiltrated	not affected	only MBs visible
6	Y	mild hypointensity	mild hyperintensity	1.6×1.3×2.1	polygonal	pure solid	marked homogeneous enhancement	Y(downward)	N	wholly infiltrated	partially infiltrated	partial invasion	only MBs visible
7	N	hypointensity	hyperintensity	0.5×0.9×0.7	round	pure solid	marked homogeneous enhancement	N	N	not affected	partially infiltrated	not affected	wholly identifiable
8	N	hypointensity	hyperintensity	0.6×0.5×0.6	round	pure solid	marked homogeneous enhancement	N	N	not affected	not affected	not affected	wholly identifiable
9	Y	hypointensity	hyperintensity	2.1×2.0×2.2	round	mixed solid-cystic	marked heterogeneous enhancement	Y(downward)	N	wholly infiltrated	wholly infiltrated	whole invasion	only MBs visible
10	N	mild hypointensity	mild hyperintensity	1.3×1.1×1.2	round	pure solid	marked homogeneous enhancement	Y(downward)	N	partially infiltrated	partially infiltrated	not affected	only MBs visible
11	Y	hypointensity	hyperintensity	1.7×1.4×1.9	elliptical	solid with small cystic change	marked homogeneous enhancement	N	N	partially infiltrated	partially infiltrated	partial invasion	not visible
12	N	hypointensity	hyperintensity	1.3×1.8×1.5	lobulated	pure solid	marked homogeneous enhancement	N	N	partially infiltrated	wholly infiltrated	partial invasion	not visible
13	N	hypointensity	hyperintensity	0.6×0.6×0.5	round	pure solid	marked homogeneous enhancement	N	N	not affected	partially infiltrated	not affected	wholly identifiable
14	Y	hypointensity	hyperintensity	2.1×2.0×2.2	round	mixed solid-cystic	marked heterogeneous enhancement	Y(downward)	N	wholly infiltrated	wholly infiltrated	whole invasion	only MBs visible
15	N	mild hypointensity	mild hyperintensity	1.8×1.4×1.6	polygonal	pure solid	marked homogeneous enhancement	N	N	partially infiltrated	partially infiltrated	partial invasion	not visible
16	N	mild hypointensity	mild hyperintensity	2.1×1.9×1.1	elliptical	pure solid	marked homogeneous enhancement	Y(upward)	N	not affected	partially infiltrated	partial invasion	not visible
17	Y	mild hypointensity	mild hyperintensity	1.8×2.0×2.2	round	pure solid	marked heterogeneous enhancement	N	N	partially infiltrated	not affected	partial invasion	not visible
18	Images N/A												
19	Y	mild hypointensity	mild hyperintensity	2.0×1.9×1.3	polygonal	pure solid	marked homogeneous enhancement	Y(upward)	N	not affected	partially infiltrated	partial invasion	not visible
20	N	hypointensity	hyperintensity	1.7×1.2×1.5	round	pure solid	marked homogeneous enhancement	Y(downward)	N	not affected	partially infiltrated	partial invasion	only MBs visible
21	N	hypointensity	hyperintensity	2.1×1.1×2.4	polygonal	pure solid	marked homogeneous enhancement	N	N	not affected	partially infiltrated	partial invasion	not visible
22	Images N/A												
23	Y	hypointensity	hyperintensity	2.4×2.3×1.6	elliptical	pure solid	marked heterogeneous enhancement	N	N	partially infiltrated	partially infiltrated	partial invasion	not visible
24	N	mild hypointensity	mild hyperintensity	1.8×1.1×1.7	elliptical	pure solid	marked homogeneous enhancement	N	N	partially infiltrated	partially infiltrated	partial invasion	not visible
25	Images N/A												

MBs, mammillary bodies; TVF, third ventricle floor; N^*^, no; Y^†^, yes; N/A^‡^, not available.

**Figure 3:**
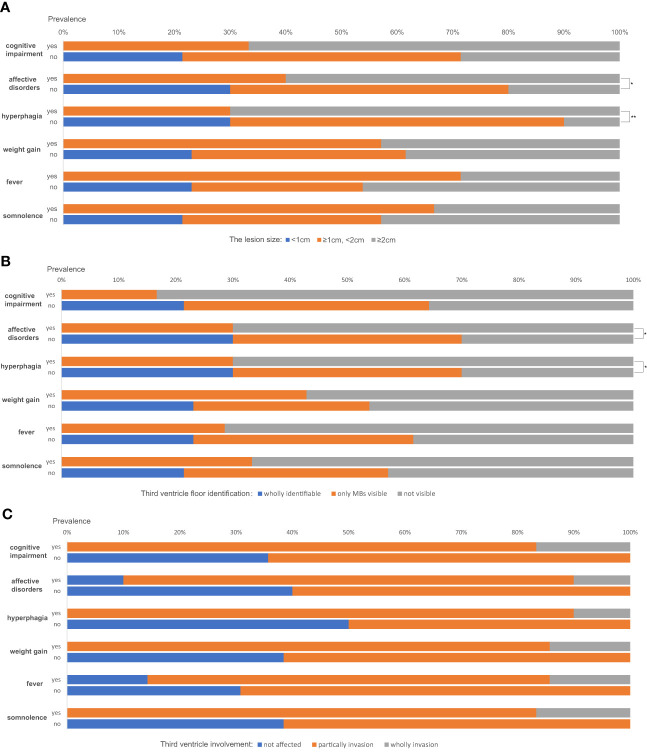
The correlations between complex hypothalamic symptoms and the lesion size **(A)**, the third ventricle floor (TVF) identification **(B)** and third ventricle involvement **(C)**. *P < 0.05, **P < 0.01.

The correlations between complex hypothalamic symptoms (including cognitive impairment, affective disorder, hyperphagia, weight gain, fever, and somnolence) and the lesion size (using the maximum diameter in any of the three planes as the variable), TVF identification, and third ventricle involvement were analyzed. The absence of any of such symptoms was only seen in cases with lesion sizes less than 1cm (see the blue bars in **Figure 3A**). Also, a larger size is a marked predictor of developing affective disorders and hyperphagia (P=0.032 and 0.005, respectively). Patients having cognitive impairment tend to have larger lesions than those who didn`t too, but the difference is not statistically significant (P=0.088). Similarly, patients with wholly identifiable TVFs showed no manifestations of such complex hypothalamic symptoms (**Figure 3B**). Those with more notable involvement of the TVFs were more likely to have symptoms of affective disorders and hyperphagia (P=0.042 for both). Generally, patients with these complex symptoms had higher degrees of the third ventricle involvement (**Figure 3C**), despite the statistic insignificance. Patient No. 9, whose third ventricle was completely invaded, developed all the six symptoms described above.

High FDG metabolism of the solitary hypothalamic lesions was found in 18-FDG-PET-CT for the nine evaluated cases, with the maximum standardized uptake values (SUVs) ranging from 5.1 to 33 (**Supplementary Table 1**). In 6/9 of the cases whose diagnoses were confirmed, the SUVs of those who had LCH were significantly higher than those who didn`t (20.87 ± 5.92 *vs.* 6.37 ± 1.17, P=0.047). Besides the primary lesions, elevated uptake of FDG was found in patient No.1 (bone marrow), No.6 (bilateral cervical, axillary, mesenteric, and retroperitoneal lymph nodes), No.18 (left upper lung, mediastinal, and bilateral pulmonary hilar lymph nodes), No.21(hepatic hilar region, skins of bilateral chest wall and axillae, muscles of the left buttock), No.22 (bilateral temporal-mandibular joint, bilateral cervical, left clavicular lymph nodes), and No.24 (bilateral submandibular, bilateral axillary, right inguinal lymph nodes and a pulmonary nodule in the right lung).

### Diagnosis

**Figure 4:**
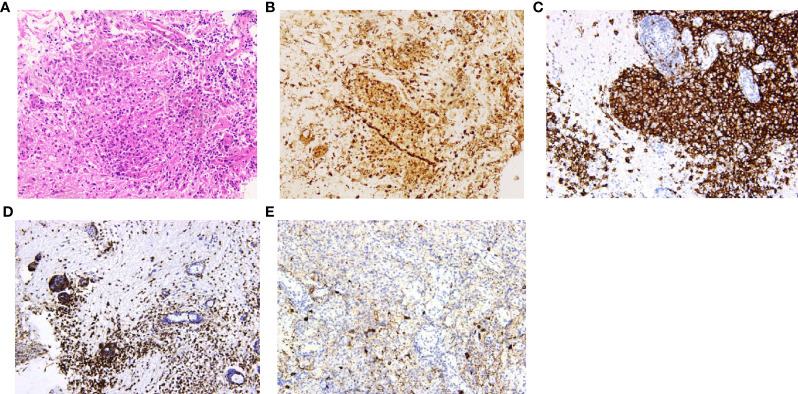
Histopathology and immunohistochemistry manifestations of Langerhans cell histiocytosis (LCH) in patient No. 10. **(A)** H&E staining reflected marked histiocyte proliferation (H&E, ×200 original magnification); Immunohistochemistry indicated clusters of various immunophenotypical markers [**(B)**: CD68+, **(C)**: CD1a+, **(D)**: leukocyte common antigen (LCA)+, **(E)**: CD138+, ×200 original magnification].

**Figure 5:**
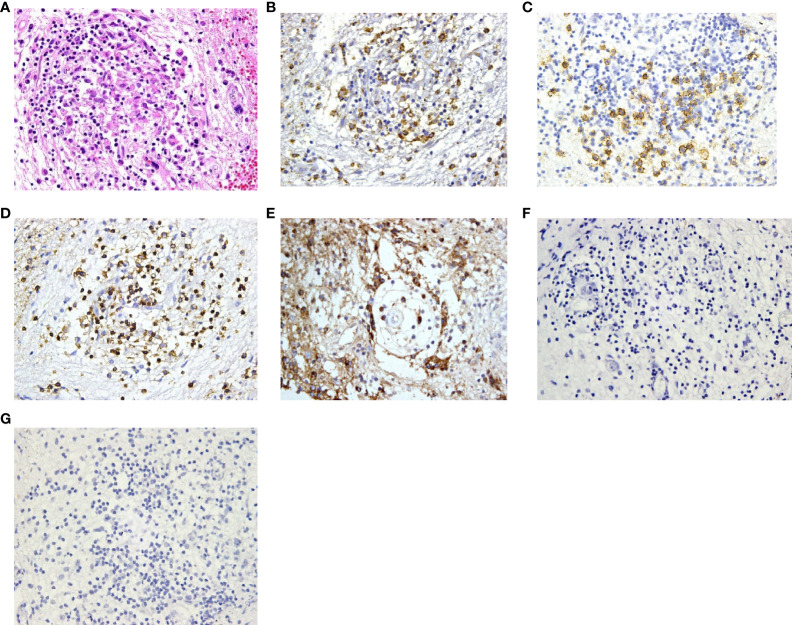
Histopathology and immunohistochemistry manifestations of hypothalamitis in patient No. 17. **(A)** Notable proliferation of lymphocyte, plasma cells and histiocyte was found in H&E staining (H&E, ×200 original magnification); Immunohistochemical analysis revealed scattered CD68 **(B)**, CD138 **(C)** and CD3 **(D)** cells, with IgG deposition **(E)**. CD20 **(F)** and CD1a **(G)** cells were absent.

Histopathological diagnoses were confirmed in 19 of the 25 patients. The mean timespan from onset to diagnosis was 34 ± 26 months. The diagnoses were various, and the most common diseases were Langerhans cell histiocytosis (7/19) and hypothalamitis (5/19). **Figures 4**, **5** show the histopathologic findings of patients No. 10 and 17, which were diagnosed with LCH and hypothalamitis separately. Marked histiocyte proliferation was found in case No. 10. Immunohistochemistry examination reflected significant abundance in CD1a cells, scattered CD68 positive results for anti-CD68 antibody (KP1) and leukocyte common antigen (LCA), and few CD138 cells. Abundant lymphocyte, plasma cells, and histiocyte were seen in case No.17. Immunohistochemical analysis revealed scattered CD68, CD138, and CD3 cells, with IgG deposition. CD20 and CD1a cells were absent. Those with hypothalamitis were treated with glucocorticoids and/or azathioprine, and post-therapeutic MRI revealed mass volume reduction. In the 20 patients receiving neurosurgical biopsy/resection or stereotactic biopsy, definite histopathological diagnosis was not achieved in 4 of them (No.1, 6, 20, and 24). Only gliosis and inflammatory cell infiltration were found, with no diagnostic immunohistochemical features. For patient No.20, two years after the surgical biopsy through craniotomy, a subcutaneous lump on the forehead gradually arose. The biopsy of the lump later suggested the diagnosis of LCH. Patient No.21 received a skin biopsy, and it turned out to be LCH. Patient No. 18, the eldest case of all, was found to have mediastinal lymph node enlargement in the chest computed tomography (CT) scan. The lymph node biopsy helped to confirm the histopathological diagnosis of low-differentiated neuroendocrine carcinoma originated from the lung. Thus, the mass of the patient’s hypothalamus was thought of as the metastatic lesion of the lung carcinoma.

### Pituitary Dysfunctions and Metabolic Disorders

**Figure 6:**
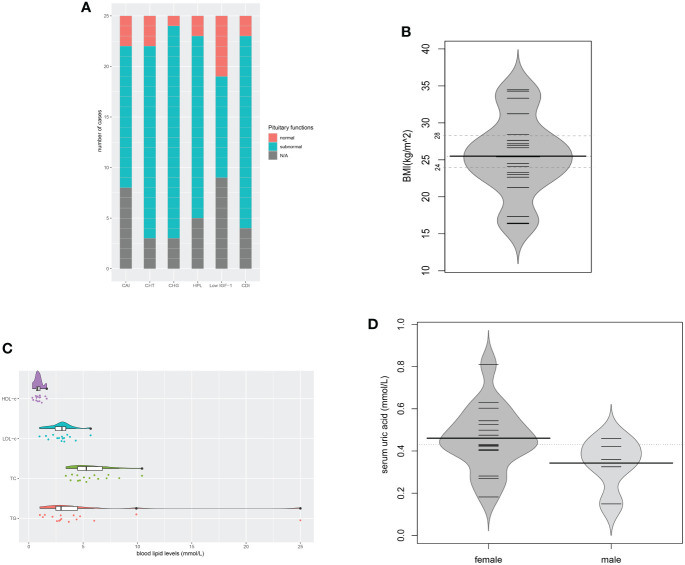
Pituitary dysfunctions and metabolic disorders. **(A)** Description of hypopituitarism of all the cases; **(B)** BMIs of the evaluated cases (n = 20); **(C)** Blood lipid levels of the evaluated cases (n = 16); **(D)** Serum uric acid levels of the evaluated cases (n = 20). CAI, central adrenal insufficiency; CHT, central hypothyroidism; CHG, central hypogonadism; HPL, hyperprolactinemia; CDI, central diabetes insipidus; N/A, not available.

In the evaluation of pituitary functions, central hypogonadism (21/22, 95.5%) and central diabetes insipidus (19/21, 90.5%) were found the two most commonly affected axes (**Figure 6A**). The prevalence of hypopituitarism of almost all different axes (including the adrenal gland, thyroid gland, gonadal glands, prolactin, and neurohypophysis) in the evaluated patients exceeded 80%. Insulin-like growth factor-1 (IGF-1) levels below the age- and gender-matched reference range were found in 10 of the 16 patients.

Metabolic evaluation in the cases revealed remarkable metabolic disorders. Pre-operation/treatment data of body mass index (BMI) were available in 20 cases (**Figure 6B**). Overweight (24≤BMI ≤ 27.9) was found in 8 (40.0%), and obesity (BMI≥28) in 5 (25.0%). Hyperlipidemia developed in 13 of the 16 evaluated cases. The levels of triglyceride (TG), total cholesterol (TC), low-density lipoprotein-cholesterol (LDL-c), and high-density lipoprotein-cholesterol (HDL-c) were 4.87 ± 5.78, 5.77 ± 1.90, 3.00 ± 1.14, 0.90 ± 0.36 (mmol/L), respectively. Among them, elevated triglyceride (TG) was found in all 13 cases. Patient No. 5 even had a TG level as high as 25.0mmol/L (**Figure 6C**). In the evaluation of glucose metabolism, 7/16 cases were proved to have diabetes mellitus (DM), 3/16 had impaired glucose tolerance (IGT). Hyperuricemia was found in 10 of the 15 females and 2 of the 5 males, with serum uric acid levels of 0.460 ± 0.156mmol/L and 0.342 ± 0.120mmol/L separately (**Figure 6D**). No onset of gout was reported. In the 12 patients with hyperuricemia, 5 cases had no elevated serum sodium level. Liver ultrasonography demonstrated hepatic adipose infiltration in 11 of the 16 evaluated patients. No history of excessive drinking was reported, nor evidence of viral/autoimmune/congenital hepatic diseases was found. Levels of the serum lipid profile, HbA1c, FBG, PBG, were not significantly related to BMI, while levels of SUA were positively related to BMI (r=0.652, P=0.003). The BMIs of those who had fatty liver infiltration were notably higher than patients who didn`t (P=0.027).

## Discussion

Previous reports had given descriptions of the manifestations of patients with hypothalamic lesions, resulting from the mass effects or the dysfunction in the regulation of endocrine disorders (insipidus diabetes, amenorrhea, sexual dysfunction), sleep, food intake, body temperature, water metabolism, emotion, which were called hypothalamic syndrome (1). Early in the 1950s, Bauer reported 60 cases with various lesions in the hypothalamus (8). Neurologic symptoms were the most common clinical manifestations (including neuro-ophthalmologic abnormalities, pyramidal tract or sensory nerve involvement, headaches, extrapyramidal cerebellar signs, and recurrent vomiting). As for the symptoms related to the hypothalamus itself, sexual abnormalities, diabetes insipidus, and psychic disturbances were the top three manifestations. Specially, precocious puberty was seen in 40% of the cases, which may result from the selection bias. However, few reports had described the symptoms of solitary hypothalamic lesions. In our case series, diabetes insipidus was the most common initial manifestation, affecting 76% of all cases, and the prevalence of polyuria and polydipsia rose to 80% later. Hypogonadism in females was another frequently reported initial symptom (in 9 of the 20 cases). In addition, with the progress of the diseases, manifestations of hypogonadism developed in all the patients over 18 years old. Other symptoms include cognitive impairment, affective disorders, food intake disorders, body weight alternations (either gain or loss), fever, somnolence, oligodipsia, lactation, neurologic symptoms, psychiatric symptoms, and cutaneous/subcutaneous involvement. Our institution mainly deals with adult patients. The youngest age of onset was ten years old in our report. That`s why precocious puberty wasn`t seen. Compared to Bauer`s report, symptoms linked to hypothalamus dysfunction were more common in our case series rather than neurological symptoms. This could be explained by the localized involvement of the hypothalamus, with the adjacent anatomic structures (such as the optic chiasm, the optic nerve, the internal capsule, the pituitary, the pituitary stalk, and the thalamus, etc.) less (or not) affected.

The lesions involving different nuclei and regions of the hypothalamus can result in related symptoms. Central diabetes insipidus mainly results from destructions of the supraoptic and paraventricular nuclei (9). In addition, the dysfunctions of the thirst center osmoreceptors lead to oligodipsia, which may cause severe hypernatremia. As diabetes insipidus was the most common initial symptom in our report, it could be inferred that a primary hypothalamus lesion often firstly affects the supraoptic and paraventricular nuclei. The susceptibility of developing hypogonadism can be another characteristic of solitary hypothalamus lesions. In our study’s evaluation of pituitary functions, central hypogonadism had the highest prevalence (95.5%) of all axes. Similarly, as one of the most frequently affected axis in sellar lesions, the previously reported prevalence of central hypogonadism could be as high as 95% in patients with sellar tumors and after surgery or radiotherapy (5). The mechanisms of hypothalamic hypogonadism are complex, including the disorders of gonadotropin-releasing hormone (GnRH) secretion/transportation, pulsatile release of luteinizing hormone (LH) and follicle-stimulating hormone (FSH), and/or hyperprolactinemia, etc. (1) Manifestations of somnolence were common in our and the previous reports (8). The mechanisms of sleep-wake cycle regulation are rather complex. The hypothalamus harbors nuclei and regions controlling sleep-wake cycles, and they communicate with other areas of the brain, especially the reticular activating system of the brain stem, to maintain a normal circadian rhythm (10). Destructions of the hypothalamus could impair normal wakefulness and arousal and lead to prolonged hours of sleep (1). Fever with no evidence of infection occurred in 7 cases. The preoptic area (POA) of the hypothalamus is thought to be the key integratory site for thermoregulation in the brain (11). The symptoms of fevers in such patients could very possibly result from the abnormal central regulation of body temperature. Affective disorders, especially irritability, were found in some of our cases. Lesions in the ventromedial nucleus have been found to be related to aggressive and even violent behaviors in animals (1). Early in 1969, Reeves and Plum described a case whose ventromedial hypothalamus was destroyed by a small neoplasm manifested rage attacks, hyperphagia, and dementia (12). Bilateral involvement of ventromedial nuclei and adjacent pathways contribute to such a triad. Cognition impairment in our reports mainly manifested as memory deterioration, which might be associated with dysfunctions of ventromedial nuclei and the communications between the hypothalamus and brain stem reticular formation and limbic system (1). Also, posteriorly the tuber cinereum lie the mammillary bodies. They include important nuclei of the memory circuit, and hypothalamic lesions affecting the fornices and the afferent input to the mammillary bodies could cause memory defects (13). Such manifestations had been widely described in Korsakoff’s syndrome, which is mainly characterized by amnesia (both retrograde and anterograde) (14). The pathology in Korsakoff’s syndrome almost always involves the mammillary bodies. Although Korsakoff’s syndrome is most frequent in alcoholics, it is also described for craniopharyngiomas affecting the third ventricle (15). It can be inferred that Korsakoff-like amnesia could be a significant determinant of memory deteriorations in our case series. Besides, disorders of the sleep-wake cycle and poor quality of sleep could increase the possibility of cognitive impairment as well (10). In addition to emotional and cognitive alterations, strange behaviors and changes in personality, even accompanied by psychotic symptoms can occur, as was in the case No. 23. Such symptoms reflect lesions affecting more diffusely the hypothalamic networks and the connectivity of the hypothalamus with the medial-basal frontal cortices and limbic system (16). Our study validates the hypothalamic lesions as a clinical model of psychiatric disturbances, similarly to the case of craniopharyngiomas developing or invading the third ventricle. Accordingly, a wider set of symptoms than the insufficiency of hormones of the endocrine axes controlled by the hypothalamus can be grouped under the term “hypothalamic syndrome” in contrast to the restricted involvement of the infundibulum-tuber cinereum.

In craniopharyngiomas, a clinical-topographical correlation between the patient’s syndrome and the anatomical structures involved by the tumor had been proven (17). Structural/functional impairment of the infundibulum-tuber cinereum complex encompassing damage of the median eminence, arcuate nucleus, and tubero-mammillary nuclei, leads to the infundibulo-tuberal syndrome. It was originally described by French authors Claude and Lhermitte in 1917 (18), including symptoms of Fröhlich`s syndrome, obesity, diabetes insipidus, and/or sleep alterations due to lesion of the histaminergic neurons in the tuberomammillary nucleus (19). Infundibulo-tuberal syndrome was found to occur in craniopharyngiomas which replaced or invaded the TVFs (17). Furthermore, injury to structures above the infundibulum and tuber cinereum level (ventromedial and dorsomedial hypothalamus and fornices) induces complex psychiatric, behavioral, or emotional alterations, memory impairment (including Korsakoff’s syndrome), abnormal body temperature, and so on. In our study, the clinical-topographical correlation was also confirmed. Hyperphagia and somnolence, as typical manifestations of infundibulo-tuberal syndrome, were only seen in cases with the TVF involvement. Besides, those with complex hypothalamic symptoms associated to cognition, mood and body temperature tended to have larger mass sizes, wider involvement of third ventricle and TVFs.

**Figure 7:**
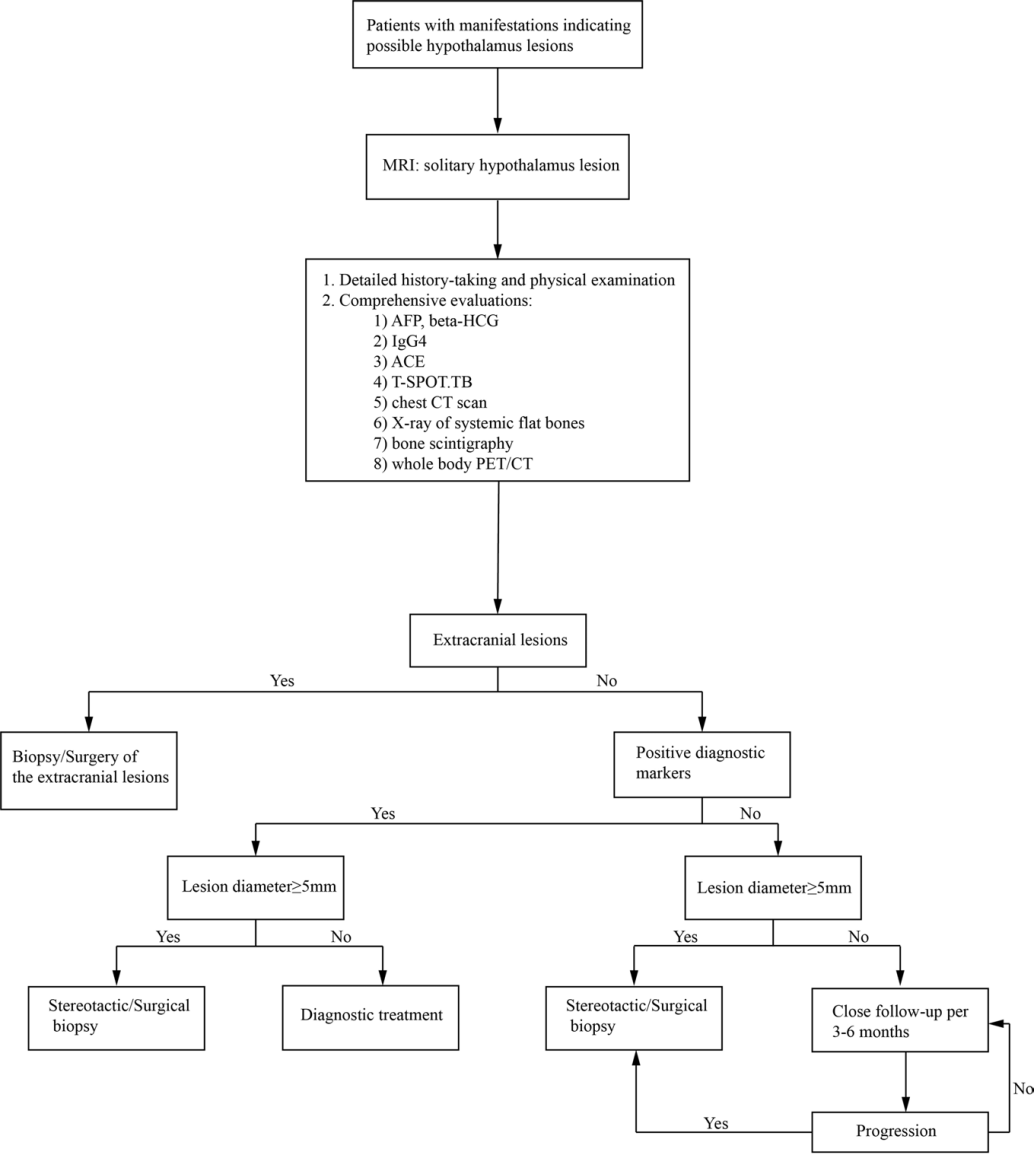
An algorithm of the diagnosis of an intracranial solitary hypothalamus lesion. MRI, magnetic resonance imaging; ACE, angiotensin-converting enzyme; T-SPOT.TB, T-cell spot of tuberculosis test; CT, computed tomography; PET/CT, Positron emission tomography-computed tomography.

The solitary hypothalamic lesion occurs rarely, and patients with different etiology could have similar clinical manifestations and MR imaging features. Final diagnoses highly depend on histopathological evidence. The surgical procedures in the hypothalamus region could be risky and challenging. Diagnostic radiation and steroid treatment are alternatives when the etiology is hard to confirm. However, the treatment can be ineffective and induce lots of complications. Only in very few case reports the pathological diagnoses were established (2–4). In our case series, stereotactic hypothalamic biopsy/surgical biopsy/surgical resection was performed in 20 patients by the most experienced surgeons in our institution. 16 of the 20 cases achieved histopathological diagnoses. Either surgical or stereotactic biopsy will be a reliable and relatively safe procedure to help to reach the pathological diagnosis of solitary hypothalamic mass. The stereotactic biopsy can be used for most hypothalamic primary lesions with diameters≥5mm. For lesions located at the bottom area of the hypothalamus or close to the pituitary stalk, a surgical biopsy is more likely to be chosen. In patients No.1, 6, 20, and 24, only gliosis and inflammatory cell infiltration were found, with no diagnostic immunohistochemical features. However, the following subcutaneous lump in patient No. 20 gave rise to the final diagnosis of LCH. Similarly, the skin lesions in patient No. 22 made the diagnosis of LCH. The lymph node biopsy in Patient No.18 helped to confirm the histopathological diagnosis of low-differentiated neuroendocrine carcinoma originated from the lung. So, the mass of the hypothalamus was thought of as a metastatic lesion. Such cases indicated that careful systemic evaluations are critical, including detailed physical examination and history-taking, evaluation of important diagnostic markers, and comprehensive radiological examinations. CT scan and whole body 18-FDG-PET-CT scan can help to find potential extracranial lesions for biopsy or surgery. Also, the multi-disciplinary workup and consultation consisting of specialties of different departments can be vital to reach the final diagnosis. Here we present an algorithm of diagnosis of a solitary hypothalamus lesion (**Figure 7**).

In our cases, the metabolic disorders were remarkable, which appeared before any etiological therapy. Previous reports seldom emphasized the problems of metabolism. The hypothalamus plays a vital role in regulating body weight by balancing the intake of food and energy expenditure and storage. Hypothalamic obesity (HO) is defined as significant hyperphagia and weight gain after any damage to the energy-controlling center of the hypothalamus (20).HO is most commonly described in the context of craniopharyngioma, and most patients with HO have large lesions or extensive involvement of the hypothalamus (21). Previous reports indicate that obesity occurs in approximately 25% of individuals with anatomically proven lesions in the hypothalamus (8, 9). The prevalence of weight gain could be as high as 90% in craniopharyngioma after surgery (21). In our reports, hyperphagia was seen in 48% (12/25) of cases, and overweight/obesity was found in 65% (13/20). The mechanisms of HO could be various, including hyperphagia, impaired energy expenditure and thermoregulation, vagally mediated hyperinsulinemia, and defective hypothalamic leptin signal transduction (1, 22). Lesions affecting the arcuate nucleus, the paraventricular nucleus, the ventromedial nucleus, the dorsomedial nucleus, and the dorsal hypothalamic area could contribute to HO, which play vital roles in the regulation of satiety and energy expenditure (23). Besides, some patients in our study presented with amenorrhea, sexual dysfunction, HO and hyperphagia. Early in 1901, Alfred Fröhlich published his famous work describing a 14-year-old boy with a large pituitary tumor presenting sexual infantilism and obesity (24). These manifestations were defined as Fröhlich’s syndrome or adiposogenital dystrophy, which featured excessive eating, obesity, pubertal delay, and hypogonadism (25). The destruction/anatomic distortion of the median eminence, where axonal endings from GnRH neurons establish their synaptic contacts with the complex capillary network of fenestrated vessels and tanycytes, could contribute to the development of Fröhlich’s syndrome (26, 27). In addition, the interrupted communication between GnRH neurons and the network of astrocytes processing the feedback metabolic information of blood energy molecules and hormones to the arcuate nucleus is also a fundamental anatomical correlate of such a functional impairment (26, 28, 29). HO has a significant adverse impact on quality of life and increases the risk of cardio- and cerebrovascular mortality. In spite of a great deal of theoretical understanding, an effective treatment for hypothalamic obesity has not been developed. The trials with GLP-1 (30, 31) and gastric bypass surgery (32) have proved to be effective. We expect more effective therapy in the future. Hyperlipidemia developed in 81.3% (13/16), and hepatic adipose infiltration in 68.8% (11/16) of the evaluated cases. Fatty liver infiltration might be induced by obesity, considering the significant difference in BMI between those with and without fatty liver. Hyperglycemia and fatty liver infiltration were also reported in the follow-up of craniopharyngioma (33). Abnormal glucose metabolism (either DM or IGT) was found in 62.5% (10/16). The disorders in lipid and glucose metabolism could be caused by HO, hypopituitarism, and disturbed central metabolic regulation. Specially, we had reported a case of hypothalamitis who presented a notable elevation of blood glucose along with the increase of the size of the lesion, and the blood glucose returned to normal after post-therapeutic size decrease of the lesion (4). For this case, it can be inferred that the hyperglycemia was mainly caused by the lesion itself, which induced severely impaired hypothalamic regulation of glucose homeostasis. The diabetes mellitus resulting from reasons like this could be defined as a new type: hypothalamic diabetes mellitus. SUA levels were elevated in 60% (12/20) patients, in which 5 cases had no elevated serum sodium level. All the patients with hyperuricaemia were asymptomatic. Aside from diabetes insipidus and the following dehydration, hyperuricaemia was generally thought to be associated with genes, obesity, gender, diet, insulin resistance, drug use, chronic kidney disease, and so on (34). Levels of SUA were positively related to BMI in our study, indicating the contribution of obesity in the development of hyperuricaemia. Besides, it has been reported that electrical stimulation of the ventromedial hypothalamus in rats can induce a rise of plasma uric acid, which possibly can be due to the acceleration of epinephrine release from the adrenal medulla (35, 36). More researches are needed to explore how serum uric acid levels are regulated by the hypothalamus.

## Conclusion

Either surgical or stereotactic biopsy will be a reliable and relatively safe procedure to help to confirm the pathological diagnosis of solitary hypothalamic mass. Metabolic disorders were severe in patients with solitary hypothalamic mass. The management of such cases should cover both the treatment of the primary disease and the endocrine and metabolic disorders. Effective therapy of the metabolism disorders related to hypothalamus destructions, especially hypothalamic obesity, are to be explored in the future.

## Data Availability Statement

The raw data supporting the conclusions of this article will be made available by the authors, without undue reservation.

## Ethics Statement

The study was approved by the ethics committee of Huashan Hospital attached to Fudan University. Written informed consent to participate in this study was provided by the participants’ legal guardian/next of kin. Written informed consent was obtained from the [individual(s) and/or minor(s)’ legal guardian/next of kin] for the publication of any potentially identifiable data included in this article.

## Author Contributions

HY, YFW, and QM designed the study. BX, QS, MH, WW, QM, BL, SZ, ZZ, YY, YL, YW, ZY, HC, LP, YFW, and HY diagnosed, treated, and followed the patients. YW and ZY analyzed the MRI images. HC reviewed the histopathology results. LP conducted the stereotactic biopsies, and YFW performed the intracranial neurosurgeries. BX, QS, and HY analyzed the data, wrote and edited the manuscript. All authors contributed to the article and approved the submitted version.

## Funding

This work was supported by the National Key R&D Program of China (2019YFA0801900), the National Project in promoting the diagnosis and treatment of major diseases by MDT, and the National Natural Science Foundation for Young Scientists of China (Grant No. 81800691).

## Conflict of Interest

The authors declare that the research was conducted in the absence of any commercial or financial relationships that could be construed as a potential conflict of interest.

## Publisher’s Note

All claims expressed in this article are solely those of the authors and do not necessarily represent those of their affiliated organizations, or those of the publisher, the editors and the reviewers. Any product that may be evaluated in this article, or claim that may be made by its manufacturer, is not guaranteed or endorsed by the publisher.
