# Supplementary material for: Successful Diagnoses and Remarkable Metabolic Disorders in Patients With Solitary Hypothalamic Mass: A Case Series Report
Source: Front Endocrinol (Lausanne). 2021 Sep 16;12:693669. doi: 10.3389/fendo.2021.693669 (PMC8481775; doi:10.3389/fendo.2021.693669)
Supplement: Supplementary file 1 [file Table_1.docx]

**Supplementary Table 1. SUVs of the hypothalamus lesion in total-body 18-FDG-PET-CT scan**

| **Patient No.** | **lesion SUV** | **diagnosis** |
| --- | --- | --- |
| 2 | 7.9 | Unknown |
| 6 | 11.5 | Unknown |
| 7 | 7.4 | hypothalamitis |
| 8 | 5.1 | germinoma |
| 10 | 14.08 | LCH |
| 18 | 6.6 | metastatic carcinoma from lung |
| 21 | 24.9 | LCH |
| 22 | 23.65 | LCH |
| 24 | 33 | Unknown |

SUVs: standardized uptake values; 18-FDG-PET-CT: 18-fluorodeoxyglucose positron emission tomography; LCH: Langerhans cell histiocytosis
